# Supplementary material for: Comparison of a long-read amplicon sequencing approach to short-read amplicons for microbiome analysis
Source: bioRxiv. 2025 Sep 5:2025.09.04.674341. Preprint. [Version 1] doi: 10.1101/2025.09.04.674341 (PMC12424978; doi:10.1101/2025.09.04.674341)
Supplement: 1 [file NIHPP2025.09.04.674341V1-supplement-1.pdf]

bioRxiv preprint doi: https://doi.org/10.1101/2025.09.04.674341; this version posted September 5, 2025. The copyright holder for this preprint (which was not certified by peer review) is the author/funder, who has granted bioRxiv a license to display the preprint in perpetuity. It is made available under aCC-BY 4.0 International license.

Supplemental Figure 1: The composition of a defined DNA community against the predicted composition, shown with genus-level classifications for each amplicon type tested, excluding StrainID. The *in silico* amplicons were derived from StrainID reads that were trimmed to the appropriate length prior to ASV calling.

Supplemental Figure 2: The number of ASVs for each taxa, compared to the expected number of ASVs and total rRNA operon copies in the reference genomes of all 8 members of the mock community for all amplicon types tested.

Supplemental Figure 3: Box and whisker plots for the proportion of reads classified at the family, genus, and species levels with the Greengenes2 database for both saliva (A) and fecal samples (B). StrainID ASVs were trimmed to include only the full-length 16S region to be compatible with the database.

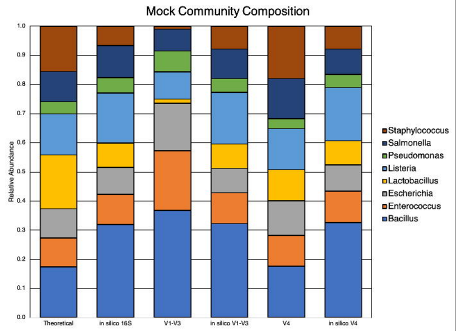
Mock Community Composition
Relative Abundance
1.0
0.9
0.8
0.7
0.6
0.5
0.4
0.3
0.2
0.1
0.0
Theoretical
in silico 16S
V1-V3
in silico V1-V3
V4
in silico V4
Staphylococcus
Salmonella
Pseudomonas
Listeria
Lactobacillus
Escherichia
Enterococcus
Bacillus

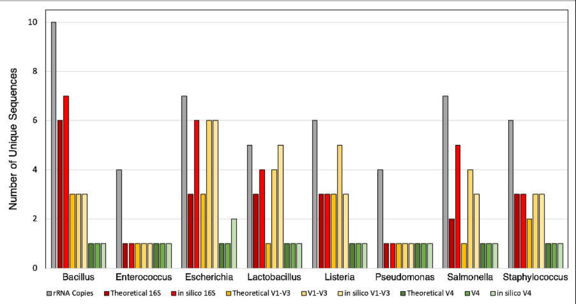

Number of Unique Sequences
0
2
4
6
8
10
Bacillus
Enterococcus
Escherichia
Lactobacillus
Listeria
Pseudomonas
Salmonella
Staphylococcus
rRNA Copies
Theoretical 16S
in silico 16S
Theoretical V1-V3
V1-V3
in silico V1-V3
Theoretical V4
V4
in silico V4

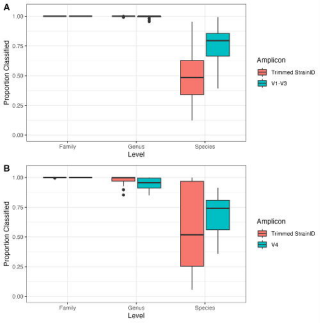
A
B
Proportion Classified
1.00
0.75
0.50
0.25
0.00
Family
Genus
Species
Level
Amplicon
Trimmed StrainID
V1-V3
V4
